# Protein complex prediction via verifying and reconstructing the topology of domain-domain interactions

**DOI:** 10.1186/1471-2105-11-350

**Published:** 2010-06-28

**Authors:** Yosuke Ozawa, Rintaro Saito, Shigeo Fujimori, Hisashi Kashima, Masamichi Ishizaka, Hiroshi Yanagawa, Etsuko Miyamoto-Sato, Masaru Tomita

**Affiliations:** 1 Institute for Advanced Biosciences, Keio University, 403-1, Daihoji, Tsuruoka, Yamagata 997-0017, Japan; 2 Systems Biology Program, Graduate School of Media and Governance, Keio University, Endo 5322, Fujisawa, Kanagawa 252-8520, Japan; 3 Faculty of Environment and Information Studies, Keio University, Endo 5322, Fujisawa, Kanagawa 252-8520, Japan; 4 Graduate School of Science and Technology, Keio University, 3-14-1 Hiyoshi, Kohoku, Yokohama, Kanagawa 223-8522, Japan; 5 IBM Research - Tokyo, 1623-14 Shimo-tsuruma, Yamato, Kanagawa, 242-8502 Japan; 6 Graduate School of Information Science and Technology, The University of Tokyo, 7-3-1 Hongo, Bunkyo-ku, 113-8656 Tokyo, Japan

## Abstract

**Background:**

High-throughput methods for detecting protein-protein interactions enable us to obtain large interaction networks, and also allow us to computationally identify the associations of proteins as protein complexes. Although there are methods to extract protein complexes as sets of proteins from interaction networks, the extracted complexes may include false positives because they do not account for the structural limitations of the proteins and thus do not check that the proteins in the extracted complex can simultaneously bind to each other. In addition, there have been few searches for deeper insights into the protein complexes, such as of the topology of the protein-protein interactions or into the domain-domain interactions that mediate the protein interactions.

**Results:**

Here, we introduce a combinatorial approach for prediction of protein complexes focusing not only on determining member proteins in complexes but also on the DDI/PPI organization of the complexes. Our method analyzes complex candidates predicted by the existing methods. It searches for optimal combinations of domain-domain interactions in the candidates based on an assumption that the proteins in a candidate can form a true protein complex if each of the domains is used by a single protein interaction. This optimization problem was mathematically formulated and solved using binary integer linear programming. By using publicly available sets of yeast protein-protein interactions and domain-domain interactions, we succeeded in extracting protein complex candidates with an accuracy that is twice the average accuracy of the existing methods, MCL, MCODE, or clustering coefficient. Although the configuring parameters for each algorithm resulted in slightly improved precisions, our method always showed better precision for most values of the parameters.

**Conclusions:**

Our combinatorial approach can provide better accuracy for prediction of protein complexes and also enables to identify both direct PPIs and DDIs that mediate them in complexes.

## Background

Recently developed high-throughput methods such as yeast two-hybrid or mass spectrometry to obtain protein-protein interactions (PPIs) have provided a global view of the interaction network [1-5]. As a PPI network grows, it becomes increasingly important to detect functional modules for understanding cellular organization and its dynamics [6]. Protein complexes are clusters of multiple proteins, and they often play a crucial part in basal cellular mechanism. Therefore, computational methods to predict protein complexes are becoming important.

There are four steps in characterizing a protein complex [7]. The first step is to identify its member proteins. The second step is to determine its topology by identifying pairs of proteins which have direct interactions. The third step is to identify DDIs that mediate these direct interactions, and the final step is to specify the complete 3D structure of the complex. Most of the previous research on computational prediction of protein complexes has focused on the first step and various methods such as MCODE, MCL, and RNSC were developed, mainly based on graph theory [8-14]. The candidate complexes predicted by these first-step methods contain a non-negligible number of false positives [14]. One of the reasons for these errors is that all of these methods just extract locally dense regions as protein complexes on the assumption that proteins in complexes are highly interconnected to each other and do not consider structural limitations of interactions in the complex. Therefore methods focusing on higher steps are also important in terms of improving accuracy of the predictions. However there are few methods focusing on the second step [15-17] and there is no effective and comprehensive method for the third or fourth step [7,15]. In the present report, we use a combinatorial approach focusing not only on the first step but also on second and third steps. Our method analyzes complex candidates predicted by the first-step methods. It searches for optimal combinations of domain-domain interactions within the candidates based on an assumption that proteins in the candidates can form a true protein complex if each of the domains is used by a single protein interaction [18,19]. This optimization problem was mathematically formulated and solved via binary integer linear programming. As a result, our method can eliminate false positives in the first-step methods, and predict the detailed DDI/PPI organization of the protein complexes (i.e. it can identify both the direct PPIs and the DDIs that mediate them in a given complex).

## Methods

### Overview of protein complex prediction

The predicted results of existing methods to predict protein complexes include significant numbers of false positives [14], because they merely extract locally dense regions of the network as protein complexes, assuming that all of the proteins in complexes are highly interconnected to each other, without considering any structural limitations against interactions within the complex.

**Figure 1:**
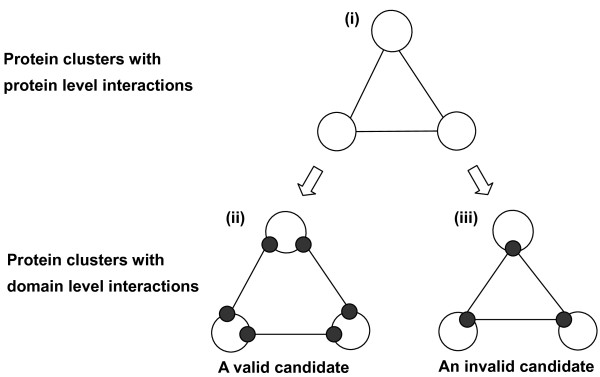
**An overview of protein complex prediction that considers the physical binding domain**. The key idea of our approach is shown in Figure 1. A densely connected protein cluster in the protein-level interaction (i) is not always the protein complex in which the member proteins can bind to each other at the same time when we consider the domain-level interactions (ii and iii); In case (ii), each protein has a sufficient number of domains to bind to each other whereas in case (iii), there are too few domains for each protein and the proteins cannot simultaneously bind to each other. Figure 2 is an overview of our method with its two main steps, extraction of protein clusters and verification of the protein clusters based on the physical binding domains.

The key idea of our approach is to eliminate the false positives by considering the exclusiveness of the binding domains. Figure 1(i) is a clique that consists of three proteins that seem to form a densely connected protein complex. However there are two primary possibilities for the actual clique if the domain-level interactions are examined. One possibility is that each protein has enough domains to bind to each other as shown in Figure 1(ii). The other possibility is that there are too few domains and the proteins cannot simultaneously bind to each other, as shown in Figure 1(iii). The latter case, which might be predicted by existing methods, is regarded as a false positive that our method can filter out.

**Figure 2:**
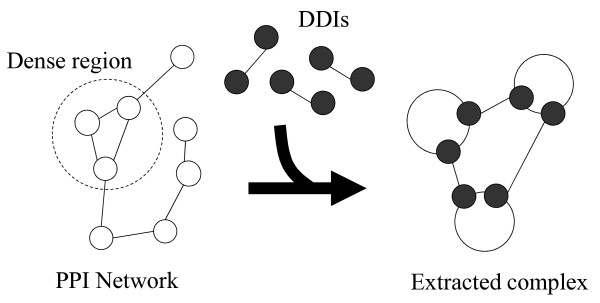
**An overview of protein complex prediction that considers the physical binding domain**. Our method mainly consists of two steps, extraction of protein clusters and verification of the protein clusters based on physical binding domains.

An overview of our analytic approach is shown in Figure 2. The prediction mainly consists of two parts, extraction of the protein clusters and verification of the protein clusters, where each PPI is mediated by the DDIs based on the exclusiveness of the binding interfaces.

First, the dense regions in the PPI network are extracted by using existing graph algorithms, assuming that nodes and edges correspond to proteins and interactions, respectively. Since proteins participating in the complex are likely to interact with each other frequently, dense regions are likely to correspond to protein complexes [8,9]. Thus, dense regions that are extracted by using existing methods can be assumed to be initial candidates for protein complexes.

Second, the initial candidates are verified by integrating the DDIs into the candidates by considering the physical binding domains based on two assumptions: (1) a protein participating in the candidate can bind to another protein within the same candidate if there is a potential DDI between these two proteins, and (2) each domain can participate in only one DDI at a time. The second assumption is based on the tendency of the binding interfaces to be exclusive [18,19], since we roughly equate a single domain with a single binding interface. Although there are cases where a single domain binds multiple domains simultaneously, we can greatly simplify our calculations by discounting those cases.

The initial candidate will be accepted as a final complex prediction if three or more proteins in the candidate are predicted to be connected by DDIs. In this way, we can consider the physical bindings in the protein complex.

### Formulation of the second step as a binary integer program

Time complexities are problematic when adopting a brute force approach to determine the physical binding domains for protein complex prediction. The most naïve approach is the enumerations of all 2^*n *^possible combination of the DDIs in a predicted protein cluster, where *n *is the total number of DDIs in the candidate protein complex. To verify the candidates much more efficiently, we formulated this step as a problem in binary integer programming. Binary integer programming is a restricted form of linear programming in which each variable in the constraints is required to be 1 or 0. Let *P*_*i*_,_*j *_∈ Ω be the potential PPI between protein *p*_*i *_and *p*_*j *_in the candidate where *P*_*i*_,_*j *_= 1 denotes the case in which the PPI actually takes place and otherwise 0. The symbol Ω denotes the set of all potential PPIs that are obtained from PPI experiments. Our objective is to maximize the number of total PPIs denoted by this equation:(1)

This maximization is rationalized by the fact that the proteins in the complexes are generally densely connected [8,9]. Then we consider two types of constraints, PPI-based constraints and DDI-based constraints. The PPI-based constraints represent the relationships between PPI variables and DDI variables. Let *D*_*i*_,_*j *_= {*D*_*i*_,_*j*__,1_, *D*_*i*_,_*j*__,2_, ... } be the potential DDIs that connect a pair of proteins, *p*_*i *_and *p*_*j*_. The value of *D*_*i*_,_*j*_,_*k *_= 1 if the pair of domains in *D*_*i*_,_*j*_,_*k *_actually interact. A PPI variable *P*_*i*_,_*j *_becomes 1 if any of the DDIs in *D*_*i*_,_*j *_is 1, as described by this equation:(2)

Domain-based constraints guarantee that each domain participates in a single DDI. Let *d*_*l *_be one of the domains in the candidate and *D*(*d*_*l*_) be a set of DDIs, where each DDI contains domain *d*_*l *_and another domain. This constraint is denoted as:(3)

**Figure 3:**
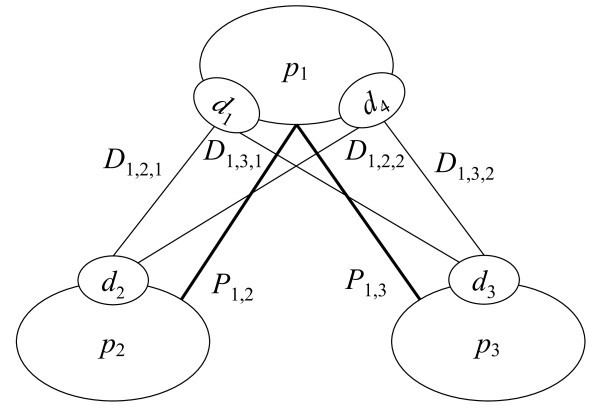
**Protein complex prediction based on PPI and DDI information**. An example of a protein complex candidate with two PPIs and four DDIs. The thick and thin lines denote PPIs and DDIs, respectively. The complex consists of three proteins namely *p*_1_, *p*_2_, and *p*_3_. The protein *p*_1 _has two domains *d*_1 _and *d*_4_. The proteins *p*_2 _and *p*_3 _have the domains *d*_2 _and *d*_3_, respectively.

For example, in the case of the candidates in Figure 3, the PPI-based constraint is *P*_1,2 _= *D*_1,2,1 _+ *D*_1,2,2 _and *P*_1,3 _= *D*_1,3,1 _+ *D*_1,3,2_. In this example, both *D*_1,2,1 _and *D*_1,3,1 _contain domain *d*_1 _but can only participate in one of the DDIs. Therefore the DDI-based constraint is *D*_1,2,1 _+ *D*_1,3,1 _≤1 and, similarly, *D*_1,2,2 _+ *D*_1,3,2 _≤1. From this, we suggest that proteins *p*_*i *_and *p*_*j *_actually interact if there are combinations of variables that satisfy *P*_*i*_,_*j *_= 1. This implies that there is exactly one *k *such that *D*_*i*_,_*j*_,_*k *_= 1 and this value of *k *denotes the domain-domain interaction *D*_*i*_,_*j*_,_*k *_which connects the protein pair. Any subgraphs that contain more than two proteins connected by more than one domain-domain interaction are assumed to be verified protein complexes.

### PPI and DDI datasets

We downloaded 35,353 yeast PPIs from the BioGrid database [20]. We used only the PPIs that were derived from mass spectrometry and two-hybrid experiments, since these PPIs represent physical interactions.

Three types of DDI datasets were used. The first dataset was taken from the iPfam database. We used the Pfam-A dataset, which contains 4,498 interactions [21]. The second and third datasets were from the InterDom database [22]. The second dataset is a reduced dataset from the entire InterDom DDI dataset using a flag that indicates a potential false positive. The flag becomes true if a DDI is derived from multi-domain protein interactions and if at least one of these conditions is satisfied: (1) the confidence score assigned by Interdom is lower than 1.5; (2) one of the domain partners is a low-occurrence domain; or (3) one of the domain partners is a promiscuous domain. We extracted DDIs whose flags were true from the entire dataset and used them as the second dataset. The third dataset of 167,516 interactions is the entire dataset that was available in InterDom. The second dataset has more confidence than the third dataset. Summaries of these datasets appear in Table 1. To evaluate how the performance of our method is influenced by DDI dataset, these datasets were further combined as (A), (A + B), and (A + C).

**Table 1:** Overview of PPI and DDI datasets

Datasets	Nodes	Edges	Source
PPI (BioGrid Release 2.0.40)	4,621	35,353	http://www.thebiogrid.org/
A: DDI (iPfam)	2,147	4,498	http://ipfam.sanger.ac.uk/
B: DDI (InterDom v2.0 : High Confidence)	2,295	14,854	http://interdom.i2r.a-star.edu.sg/
C: DDI (InterDom v2.0 : All)	3,990	167,516	http://interdom.i2r.a-star.edu.sg/
DDI (A + B)	3,382	18,207	-
DDI (A + C)	4,483	169,737	-

The column 'Datasets' indicates the name of each dataset. Numbers of nodes included in each dataset are shown under 'Nodes' and numbers of edges included in each dataset are shown under 'Edges'. 'Source' indicates the source of each dataset. PPI dataset release 2.0.40 was downloaded from www.thebiogrid.org. The DDI dataset was from ipfam.sanger.ac.uk (release 20.0), and from http://interdom.i2r.a-star.edu.sg/ (v2.0).

### Known complex datasets

To evaluate prediction performances of protein complexes, we used 763 known yeast protein complexes from the EMBL database http://yeast-complexes.embl.de/ because it is the most comprehensive yeast protein complex database.

### Uncharacterized proteins

The Gene Ontology annotations that were downloaded from ftp://ftp.ncbi.nlm.nih.gov/gene/DATA/ on October 28, 2008, were used to find proteins whose functions are unclear. Proteins that have "GO:0003674" or "GO:0005554" for their ID are regarded as uncharacterized proteins, since they indicate "molecular function unknown" in the Gene Ontology annotations.

### Parameters for prediction algorithms

We used two existing algorithms, MCL and MCODE, to detect dense regions in given PPI networks as a first step for our method. Clustering coefficients were also calculated to detect dense regions. We selected configurable parameters optimized for precision for MCODE and the clustering coefficient (Table 2). For MCL, we selected a value optimized for recall, because it showed the same degree of precision at any inflation value in our experiments.

**Table 2:** Configurable parameters for each algorithm and their optimal values

Algorithm	Parameter	Optimal Value*
MCL	Inflation	3.6

MCODE	Include Loops	FALSE
	Degree Cut-off	2
	Haircut	TRUE
	Fluff	FALSE
	Node Score Cut-off	0
	K-Core	2
	Max. Depth	100

Clustering Coefficient	-	0.4

* For MCODE and clustering coefficient, the values are optimized for precision. For MCL, the inflation parameter is optimized for recall, because it showed the same degree of precision at all inflation values.

### Evaluation of the predictions

To evaluate the predictions, precision recall analysis and functional enrichment analysis were used. The precision and recall of each method were computed according to these equations:(4)(5)

where "predicted", "known", and "matched" are the numbers of predicted protein complexes, known complexes, and predicted protein complexes that match with known complexes, respectively. Unlike conventional prediction problems, predicted complexes rarely match perfectly with known complexes. We therefore used the matching criterion *V *as defined by Bader et al. to evaluate the overlapping protein components of two complexes:(6)

where *N*_*p *_and *N*_*k *_are sets of proteins in a predicted complex *P *and a known complex *K*, respectively. We regarded a predicted complex as matching with a known complex if the overlapping criterion was greater than the threshold of 0.25, which was taken from the work of Chua et al. [13]. In this evaluation approach, multiple predicted complexes may match the same known complex.

To investigate the enriched level of specific protein function in each predicted complex, we calculated the ratio of the protein pairs that have the same function for each predicted result with our method. We regarded a protein pair as having the same function if their molecular functions are the same in the Gene Ontology annotations.

## Results

### Protein complex prediction and its performance

In our approach, protein complexes are predicted by combining a graph algorithm to find dense regions followed by a verification of the detected protein clusters. The results of protein complex prediction are summarized in Table 3. These protein complexes were predicted with the optimized parameters shown in Table 2. For comparison, the results without verification are also shown. The numbers of verified complexes with our approach were smaller than those predicted by MCL, MCODE, or clustering coefficients since our approach filters the initial candidates predicted by these algorithms by considering physical binding domains. Our method reduced the number of candidates by 95% to 60% from the initial candidates. However, the reduction rates decreased as the number of DDIs used for verification increased.

**Table 3:** Summary of protein complex predictions

Algorithm/Verification	Original output of algorithm*		Verified with DDI (A)		Verified with DDI (A + B)		Verified with DDI (A + C)	
	
	Numbers	Average size	Numbers	Average size	Numbers	Average size	Numbers	Average size
MCL	487	4.49	12	4.17	25	4.56	42	4.43

MCODE	90	5.87	11	5.00	23	5.35	34	5.71

Clustering Coefficient	434	8.23	47	4.55	104	5.25	178	5.88

* Original results of each algorithm that are not verified with the physical binding domain

As the complex size increases, the number of interactions among the member proteins in the complex may also increase. Such large complexes require many domains to bind to each other simultaneously if the bonding capability of the domains is limited. In other words, a cluster that contains many proteins is unlikely to have all of the possible interactions simultaneously active because the ability of each protein to bind is limited by the binding interfaces [18]. Therefore, it is more unlikely that larger complexes will be verified by our approach since it assumes that each domain can participate in only one DDI at a time. In fact, both the maximum size and the average size of complexes that are predicted by our method are smaller than those predicted by existing methods (Table 1, Additional File 1), indicating that our approach is more successful with smaller complexes.

**Figure 4:**
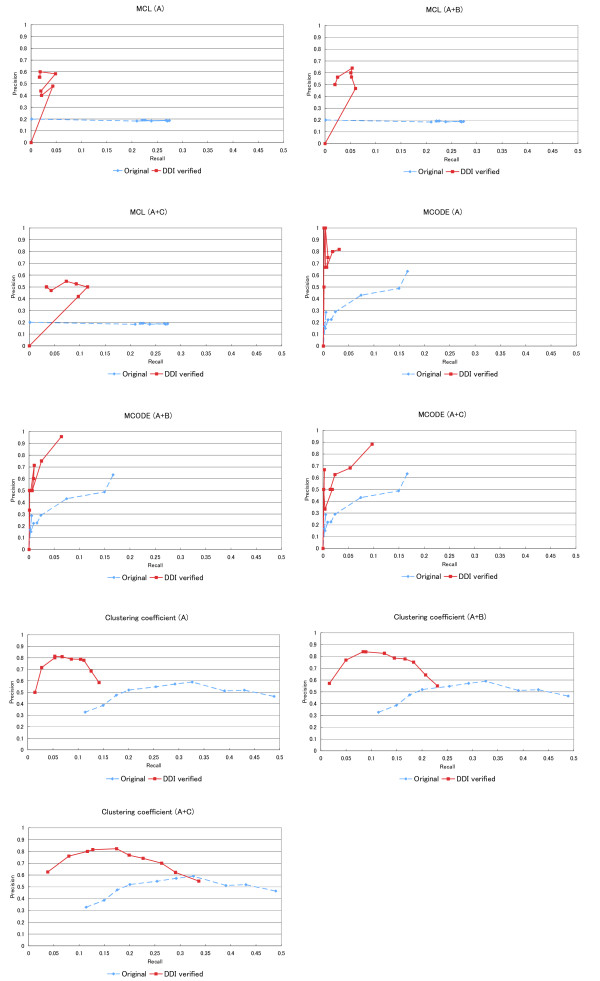
**Performance of existing algorithms and our method for the prediction of protein complexes**. Each graph is a precision-recall graph in which the vertical axis indicates the precision and the horizontal axis indicates the recall. Red lines (denoted as "DDI verified") show the results of our method and blue dotted lines indicate the results of existing algorithms (either MCL, MCODE or clustering coefficient, denoted as "Original"). Each algorithm was executed with three DDI data sets, DDI dataset (A), DDI dataset (A + B) and DDI dataset (A + C). The inflation parameter, which is only a configurable parameter in MCL, was changed from a minimum value to 1.2 to 4.8 in steps of 0.6, and MCL was also executed at a maximum value 5.0. The Node Score Cut-off parameter, which is the most influential parameter in MCODE, was changed from 0.0 to 1.0 in steps of 0.1. The threshold in the clustering coefficient was changed from 0.1 to 1.0 in steps of 0.1.

Figure 4 shows the performances of the three existing algorithms (MCL, MCODE, and clustering coefficient) and when combined with our verification method, where the x-axis is the recall and the y-axis is the precision. Both values change depending on the parameters (as described below). The performances of the three algorithms (MCL, MCODE, and the clustering coefficient) are shown as "Original" and the performances of our approach are shown as "DDI verified". We tested this procedure for three datasets, (A), (A + B), and (A + C). We changed the configurable parameters of each algorithm as shown in Table 2 to investigate their effects on the performance. The inflation parameter in MCL was changed from a minimum value to 1.2 to 4.8 in increments of 0.6, and MCL was also executed at the maximum value of 5.0. The threshold for the clustering coefficient was changed from 0.1 to 1.0 in steps of 0.1. The node score cutoff parameter, which is the most influential parameter in MCODE, was changed from 0.0 to 1.0 in steps of 0.1.

On average, our approach was twice as precise as the existing algorithms. Although configuring parameters for each algorithm resulted in slightly improved precision, most of the precision values remained lower than the precision of our approach. Our approach showed better precision with all parameter values except when the number of predicted candidates was 0. In contrast, the recalls of our approach were lower than those of the existing algorithms (31% for the existing algorithms on average). In fact, our approach drastically reduced the number of candidates (87% of the candidates). However, the reduction for recall was comparatively small (a 69% reduction). Specifically, the recall reduction of our method applied after performing MCL analysis (Inflation = 3.6) was only 80%, whereas the reduction rate of candidates was 98%. In addition, the recall of our approach improved as the number of DDIs used for the verification increased (Additional file 2). In contrast, the precision of our method was almost constant, regardless of the number of DDIs used.

**Figure 5:**
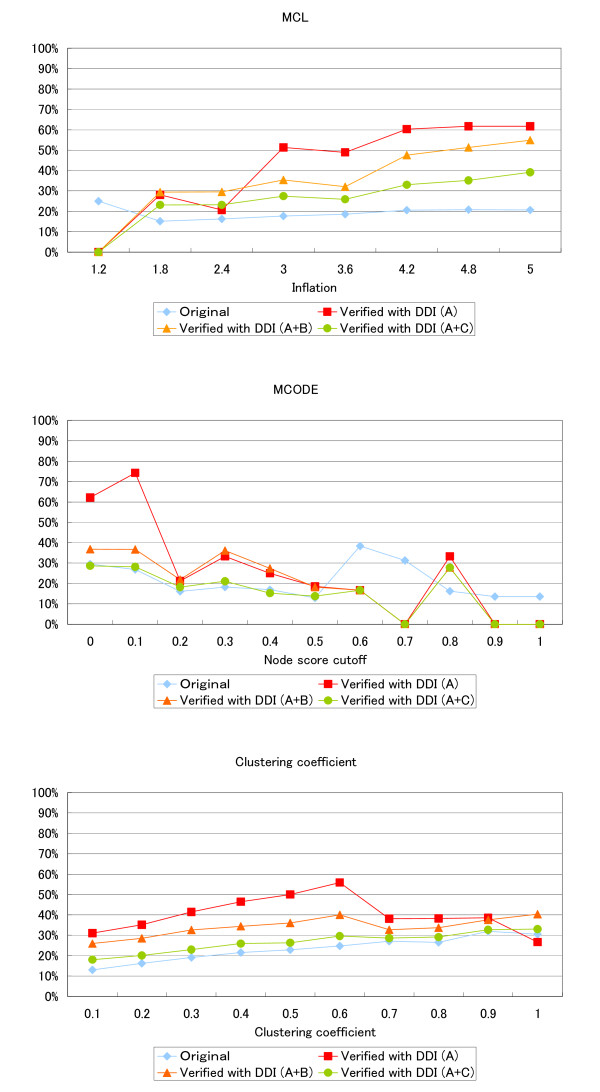
**Rate of protein pairs having the same functions in the predicted complexes**. Each graph represents the ratio of the protein pairs with the same function in each output of the methods. The vertical axis is the ratio and the horizontal axis is the configurable parameters for each method. The red, orange, and green lines (denoted as "DDI verified") indicate the results of our method and the blue lines indicate the results of the prior algorithms (MCL, MCODE, or clustering coefficient, denoted as "Original").

Proteins in a reliable protein complex are shown to share the same function and thus the functional identities of proteins in the predicted complexes may be an alternative index to assess the reliability of predictions [23]. Figure 5 shows the ratio of the pair of proteins that share the same function in each output of the methods where the x-axis is the configurable value of each method. Our approach had a better ratio than the existing methods for most parameters with all of the datasets (A), (A + B), and (A + C). Particularly with the optimized parameters shown in Table 2, our approach showed more than 25% better performance than existing methods for DDI set (A). The average performance was best with dataset (A), second with dataset (A + B), and third with dataset (A + C), which seems reasonable since the confidence of the datasets was ranked in the same order.

### Estimation of false negative rates of our method

Although the assumption that a single binding domain can participate only in a single DDI at a time is used for the simplification of the calculations for the predictions, this may result in overlooking of some of the complexes in which a single domain does bind to multiple domains at the same time, and these complexes would be the part of false negatives. To infer how often such overlooking occurs, the rate of false negatives among true positives of existing methods was calculated (Table 4). Since our method works as a filter for existing methods, we calculated the number of known protein complexes which were predicted by existing methods but were missed by our method to estimate the "net" false negatives of our method. Also since we want to estimate the rate of the overlooking by the assumption, we subtracted the number of complexes which have no DDI annotations from the number of "net" false negatives (Some known complexes were missed by our method because of the lack of DDI annotations.). Our method combined with MCODE showed the best performance among the three existing methods in terms of false negative rate, and our method combined with MCL showed the worst result. All algorithms had their best performance with DDI dataset (A + C). This means there is a tendency that more DDI data lowers the ratio of false negatives. In summary, the ratio of false negatives, part of which may be due to the assumption, ranged from about 20% to 45%.

**Table 4:** Number of false negatives for each method

	Number of false negatives of our method	Number of false negatives of existing methods	α:Number of "net" false negatives caused by the assumption *	β:Number of true positives of existing methods	Ratio (α/β)
Clustering coefficient verified with DDI (A)	683	514	102	249	41.0%

MCL verified with DDI (A)	726	554	76	209	36.4%

MCODE verified with DDI (A)	739	636	58	127	45.7%

Clustering coefficient verified with DDI (A + B)	636	514	76	249	30.5%

MCL verified with DDI (A + B)	722	554	85	209	40.7%

MCODE verified with DDI (A + B)	714	636	42	127	33.1%

Clustering coefficient verified with DDI (A + C)	590	514	51	249	20.5%

MCL verified with DDI (A + C)	707	554	78	209	37.3%

MCODE verified with DDI (A + C)	689	636	27	127	21.3%

*Number of "net" false negatives indicates known protein complexes which were predicted by existing methods but were missed by our method, since our method works as a filter for existing methods. Because some known complexes were missed by our method for the lack of DDI annotations, we calculated number of net false negatives caused by our assumption, which indicates the number of the net false negatives that have DDI annotations (See text).

### Functional analysis of predicted protein complexes

**Figure 6:**
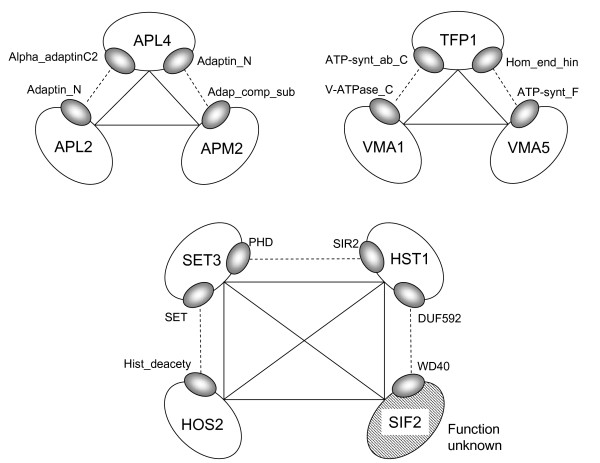
**Examples of predicted complexes**. Solid lines indicate PPIs and dotted lines indicate DDIs. The upper examples consist of three proteins and four domains. The lower example consists of four proteins and six domains. A protein with diagonal lines indicates a protein with unknown function.

A total of 233 protein complexes were predicted with optimized parameters with our approach. Among them, three complexes fully matched known complexes: pyruvate dehydrogenase, which consists of PDB1, LPD1, LAT1, PDA1, and PDX1; the COPI complex, which consists of SEC28, SEC27, SEC26, COP1, SEC21, RET3 and RET2; and the DNA polymerase alpha-primase complex, which consists of POL1, PRI2, POL12 and PRI1. The other three examples of predicted complexes are shown in Figure 6. The first example consists of three proteins and a part of a known protein complex, AP-1 adaptor complex. AP-1 is a clathrin adaptor complex that is a major structural component of clathrin coated vesicles functioning in clathrin coat assembly and cargo selection [24,25]. The second example consists of three proteins and part of a known complex, the vacuolar proton-transporting V-type ATPase V1 domain, which functions in the acidification of intracellular compartments in eukaryotic cells [26,27]. The function of the last complex is currently unclear.

Functional prediction of uncharacterized proteins is possible from the prediction of protein complexes, because proteins in a complex are likely to share the same function. In the results of our predictions, 64 complexes contain uncharacterized proteins. The four-protein complex shown at the bottom of Figure 6 contains an uncharacterized protein, SIF2. We suggest that SIF2 has a protein transporter activity function, because the other proteins in the complex have the same function. Complexes that contain a single uncharacterized protein and other characterized proteins that have the same function are shown in Table 5. The uncharacterized proteins may have the same function as the other proteins in the complexes.

**Table 5:** Complexes that contain an uncharacterized protein

Protein Complex Members	Uncharacterized Protein	Function of other members
APL1, APS2, APM4, APL3	APM4	protein transporter activity
SWM1, CDC27, CDC16, CDC23	SWM1	protein binding
SKP1, DAS1, YLR352W	YLR352W	protein binding
PWP2, NOP14, MPP10	MPP10	snoRNA binding
PWP2, UTP18, NOP58	NOP58	snoRNA binding

Complexes that include a single uncharacterized protein and other characterized proteins having the same function are shown. The column 'Members' indicates the list of protein names in each protein complex. The column 'Uncharacterized Protein' indicates the name of uncharacterized protein included in each protein complex. The column 'Function of other members' indicates the function of the other characterized proteins in each protein complex.

## Discussion

We introduced a combinatorial approach for the prediction of protein complexes focusing not only on determining member proteins in complexes but also on the DDI/PPI organization of the complexes by integrating our newly developed method with existing methods. Our method allows us to identify both direct PPIs and DDIs that mediate them in a given complex. As a result of the identification, our method can eliminate false positives in the first-step methods and can provide more accurate predictions. Also for an efficient prediction, we formalized the protein complex prediction problem by considering the physical binding domain as a binary integer programming problem so that the heuristic approaches for integer programming can be applied if the computational complexity is problematic [28,29]. Although the assumption that each binding domain is exclusive resulted missing some of the complexes in which a single domain binds to multiple domains at the same time, the restriction allows for an efficient formation of the problem. The rate of false negatives related to the assumption was at most 45.7%, but it was reduced to 20.5% with the largest DDI dataset.

Our approach predicted protein complexes with about twice the accuracy of the original output of the existing methods, and our approach always showed better precision for all of the values of the configurable parameters except for the point where the number of predicted candidates was 0. Also, our approach showed better concordance rate of the functions of the protein pairs compared to existing methods. Particularly with the optimized parameters, our approach showed more than 25% better performance than existing methods for the DDI dataset with the highest confidence.

Although the recall of our approach was lower compared to the existing methods, it improved as the number of DDIs used for verification was increased. Thus, we believe that the recall of our approach will be improved as the number of available DDIs is increased. The number may be increased not only by biochemical experiments but also by computational predictions. For comparison, Katia et al. developed a prediction method for DDIs with a parsimony approach that economizes as much as possible on the use of DDIs [30]. They formulated the problem as a linear program for which the objective function is to minimize the number of DDIs necessary to justify the underlying PPIs. There are also some computational methods to predict DDIs that could enhance the results of our approach [30-33].

To predict protein complexes, several methods employ algorithms to detect densely connected regions in a PPI network. However, the average density of real protein complexes is not particularly high. For example, the density of protein complexes in yeast is around 0.55 [14]. Thus, the extraction of dense regions in the interaction network is not sufficient for accurate predictions of the protein complexes, and pre- or post-processing of the interaction network must be combined with these graph methods.

There are several methods to extract a high confidence network from the PPI network by pre-processing [34,35]. These methods should also be useful for predictions. For example, Chua et al. filtered a PPI network with a value called the FS weight prior to protein complex prediction and improved the accuracy of their predictions [13,35]. Moschopoulos et al. developed a tool called GIBA that provides a post-processing capability for individual filters or combinations of 4 different heuristic filters and this also improved the accuracy of the predictions [36]. In contrast, our method can be used for post-processing, and it can also be combined with other methods to predict protein complexes more accurately. A key difference between our method and these other methods is that our method not only improves the accuracy of the predictions, but also reveals the organization of the protein complex including the DDIs that mediate the PPIs. Protein complexes are predicted more accurately by our method and reflect the structural characteristics of the complexes in the cells and may provide deeper insights into how proteins are organized to function in the cells.

## Conclusions

We introduced a new approach for the prediction of protein complexes. It provides both accurate predictions of protein complexes and deeper insight into each protein complex by identifying the direct PPIs and DDIs that mediate the complexes.

## Abbreviations

PPI: protein-protein interactions; DDI: domain-domain interaction.

## Authors' contributions

YO conceived the methodology, developed the verification method for the interactions of the PPIs considering the DDIs in the given complexes, and drafted the manuscript. RS developed the mapping modules from given proteins to their domains and DDIs and helped to draft the manuscript. SF supported the design of the methodology. HK helped formulate the verification problem as a binary integer programming problem. MI was involved in the biological aspects of the project. HY supervised the project. EMS was also involved in the biological aspects of the project and supported the design of the methodology. MT supervised the project. All authors read and approved the final manuscript.

## Supplementary Material

Additional file 1 **Size distribution of the predicted protein complexes**. Each graph represents a distribution of the protein complex sizes in which the horizontal axis indicates the size of the protein complexes and the vertical axis indicates the number of protein complexes for each size. Distribution graphs for MCL, MCODE, and clustering coefficient are shown. Each graph includes the result of existing algorithms and our method for all three types of DDI datasets, (A), (A + B), and (A + C). Each algorithm ran with the optimized parameters shown in Table 2. Click here for file

Additional file 2 **Performance of existing algorithms and our method with three types of datasets**. Each graph is a precision-recall plot in which the vertical axis shows the precision and the horizontal axis shows the recall. Precision-recall graphs for MCL, MCODE and clustering coefficient. Each graph includes the results of existing algorithms and our method for all three types of DDI datasets, (A), (A + B), and (A + C). Each algorithm used the optimized parameters shown in Table 2. Click here for file
