# Supplementary material for: Protein complex prediction via verifying and reconstructing the topology of domain-domain interactions
Source: BMC Bioinformatics. 2010 Jun 28;11:350. doi: 10.1186/1471-2105-11-350 (PMC2905371; doi:10.1186/1471-2105-11-350)
Supplement: Additional file 1 — Size distribution of the predicted protein complexes. Each graph represents a distribution of the protein complex sizes in which the horizontal axis indicates the size of the protein complexes and the vertical axis indicates the number of protein complexes for each size. Distribution graphs for MCL, MCODE, and clustering coefficient are shown. Each graph includes the result of existing algorithms and our method for all three types of DDI datasets, (A), (A + B), and (A + C). Each algorithm ran with the optimized parameters shown in Table 2. [file 1471-2105-11-350-S1.DOC]

**Additonal file 1. Ozawa Y. *et al.***

***.***
