# Supplementary material for: Protein complex prediction via verifying and reconstructing the topology of domain-domain interactions
Source: BMC Bioinformatics. 2010 Jun 28;11:350. doi: 10.1186/1471-2105-11-350 (PMC2905371; doi:10.1186/1471-2105-11-350)
Supplement: Additional file 2 — Performance of existing algorithms and our method with three types of datasets. Each graph is a precision-recall plot in which the vertical axis shows the precision and the horizontal axis shows the recall. Precision-recall graphs for MCL, MCODE and clustering coefficient. Each graph includes the results of existing algorithms and our method for all three types of DDI datasets, (A), (A + B), and (A + C). Each algorithm used the optimized parameters shown in Table 2. [file 1471-2105-11-350-S2.DOC]

**Additional file 2. Ozawa Y. *et al.***
